# Prevalence of sub-optimal feeding practices and associated factors in very low birth weight infants admitted to the special care unit, Kawempe Hospital in Uganda

**DOI:** 10.3389/fped.2025.1558163

**Published:** 2025-06-05

**Authors:** Lucy Amaniyo, Benbella Dektar, Victoria Nakibuuka, Jolly Nankunda, Robert Opoka, Sarah Kiguli

**Affiliations:** ^1^ Department of Pediatrics and Child Health, School of Medicine, Makerere University College of Health Sciences, Kampala, Uganda; ^2^ Department of Pediatrics and Child Health, Mulago Specialized Referral Hospital, Kampala, Uganda; ^3^ Department of Monitoring, Evaluation, and Economic Analysis, Millennium Challenge Corporation, Washington, DC, United States; ^4^ Department of Pediatrics and Child Health, St. Francis of Raphael Nsambya Hospital—Nsambya Hill, Kampala, Uganda

**Keywords:** sub-optimal feeding, growth velocity, early weight change, very low birth weight, special care, Uganda

## Abstract

**Background:**

Over 60% of Very Low Birth Weight (VLBW) infants worldwide are born in Sub-Saharan Africa and South Asia. VLBW infants are born nutritionally disadvantaged, as they are suddenly and prematurely moved from a nutrient-rich to a nutrient-deficient environment. Therefore, appropriate feeding during the early neonatal period is essential for the survival and growth of VLBW infants admitted to the special care unit of a Ugandan tertiary referral hospital.

**Methods:**

We conducted a prospective cohort study among VLBW infants admitted to the Special Care Unit at Kawempe National referral hospital in Kampala, Uganda. Stable infants with no gross abnormalities or in need of resuscitation were recruited on day two of life and followed up until day seven or to discharge or death, whichever came first. Data were collected on socio-demographic and clinical characteristics, including birth weight initiation of enteral feeds, type of feeds received, and method of feeding. Observed feeding practices were compared to recommended VLBW feeding standards for appropriateness. Infants were followed up until day 7 of life. Logistic regression analysis was used to determine the factors associated with sub-optimal feeding.

**Results:**

A total of 370 VLBW infants, with a mean gestational age of 32 weeks were enrolled. Overall, 333 (90%) had sub-optimal feeding and this was significantly associated with a sub-optimal rate of early weight change (growth velocity) [OR = 6.81, 95%, CI (2.74 to 16.97)]. Factors associated with sub-optimal feeding included: early initiation of enteral feeds [AOR = 11.03, 95%, CI (1.34 to 90.77)] and low scores on social support scale for the mother [AOR = 2.78, 95%, CI (1.14 to 6.82)].

**Conclusions:**

There is a very high prevalence of sub-optimal feeding practices for VLBW infants in this population. This calls urgent need for improved feeding practices, including early enteral feeding. Future studies should explore the long terms effects of this early sub-optimal feeding practices on growth and development within the first 48 h, barring contraindications.

## Background

From a global perspective, it is estimated that over 20 million infants are born with low birth weight annually, with a significant 20% being Very Low Birth Weight (VLBW) infants (1, 2). The majority of these infants, over 60%, are born in middle- and low-income countries, including sub-Saharan Africa (SSA) and South Asia, with a prevalence of 13% reported in these regions (1, 3). However, these figures are likely underestimated, as more than 66% of infants in SSA are not weighed at birth (3).

VLBW infants face significant nutritional challenges that can lead to severe health complications during their hospital stay. The epidemiology of these infants has been the subject of several recent investigations that clearly articulate case definitions and explore associated demographic, clinical, and socioeconomic factors. Notably (4), many employ standardized definitions for low and very low birthweight, specifically, classifying infants with birthweights below 2,500 g and 1,500 g, respectively—as a foundation for their epidemiological analysis of geographic and racial disparities. This explicit definition not only underpins their statistical modeling but also facilitates the comparison of incidence rates across different populations, thereby reinforcing the significance of uniform criteria in epidemiologic studies.

The first 1,000 days of a child's life are recognized as pivotal for development, making nutritional adequacy vital for influencing long-term health outcomes (5). Nutritional deficiencies during hospitalization can predispose these infants to chronic diseases later in life, including metabolic syndrome and developmental delays (6). For example, LBW infants, if not adequately nourished in the neonatal period, face a higher risk profile that includes increased rates of obesity and cardiovascular issues in adulthood (6).

Sub-optimal feeding practices are defined as those that do not align with established guidelines for nutrition and child-rearing, contributing to adverse health outcomes, particularly in vulnerable populations such as pre-term or low birthweight infants (1). Practices include delayed initiation (<48 h), insufficient caloric intake (<150 kcal/kg/day), or inconsistent feeding practices (1).

The unknown burden of sub-optimal feeding among VLBW infants in Uganda is a significant concern. Moreover, the national prevalence of VLBW infants is estimated at 12% (7). The Special Care Unit (SCU) at Kawempe Hospital reports that 51% of all annual admissions are VLBW infants, and tragically, 26% of these infants do not survive (8). Delayed initiation of feeds is associated with poor post-discharge growth and delay in regaining birth weight, as evidenced by studies in Uganda (9–11). Furthermore, VLBW infants receiving sub-optimal feeds face a higher risk of mortality, even post-discharge (12).

In addition, sub-optimal feeding practices have been associated with broader implications, such as increased vulnerability to infectious diseases and chronic health conditions. Inadequate breastfeeding practices can lead to lifelong health implications, including cognitive deficiencies and poor academic performance (13). Similarly, research in various regions, including India and Africa, has demonstrated that caregivers often engage in suboptimal feeding due to a lack of knowledge or resources, contributing to cycles of malnutrition and poor health outcomes for children (14, 15).

Low birth weight is a critical determinant of perinatal survival, infant morbidity, and mortality. It is the most important predictor of infant mortality, particularly within the first month of life (3, 11). VLBW infants are approximately 13 times more likely to die than heavier infants (10, 11). VLBW contributes to 10% of total infant mortality rates (7) and 26% of total deaths in the Special Care Unit at Kawempe Hospital annually (8). A study in the SCU found a post-discharge mortality rate of 19.5% among VLBW infant (16). In the long term, sub-optimal feeding of VLBW infants has a significant impact on neonatal and infant mortality at a population level (1, 17).

Upon birth, VLBW infants experience a nutritional emergency, transitioning from a nutrient-rich to a nutrient-deficient environment. This nutritional disadvantage continues post-discharge, predisposing these infants to hypothermia, hypoglycemia, electrolyte imbalances, infection, susceptibility to childhood illness, lower chances of child survival, long-term physical and mental deficiencies, cerebral palsy, and behavioral, learning, and psychosocial problems during childhood (3, 9). Sub-optimal feeding in VLBW infants increases mortality, delays birth weight recovery, and raises risks of complications like respiratory distress and necrotizing enterocolitis (9, 10, 18). Long-term impacts include delayed growth, malnutrition, and neurodevelopmental issues (19, 20). Delaying initial feeds by 48 h is significantly linked to poor growth and prolonged weight recovery (21).

In the SCU at Kawempe Hospital, enteral feeds are typically started on days 2–4 of life after the infant has tolerated a trial feed of 1–2 mls. Feeds are not calculated per kg of body weight and are rarely initiated on day one of life. The amount of feed an infant receives is determined by the attending doctor, who bases the decision on various factors like the clinical condition and tolerance of feeds. Feeds are gradually increased by adding one ml to the feeds given every 2 h daily until discharge. Infants are discharged at 10 mls of enteral feeds 2 h or earlier if their clinical condition permits it. However, it was noted that infants admitted to the SCU Kawempe Hospital receive sub-optimal feeds. The proportion of infants receiving sub-optimal feeds, their associated factors, and their effect on the growth velocities of these VLBW infants admitted to SCU, Kawempe Hospital, had yet to be scientifically studied. This study, therefore, sought to fill this knowledge gap by estimating the prevalence of sub-optimal feeding practices among very low birth weight infants admitted to the special care unit, Kawempe Hospital with the aim of enhancing the understanding of sub-optimal feeding challenges faced by VLBW infants within tertiary care units in settings similar to Kampala, Uganda.

Previous studies have demonstrated the detrimental effect of sub-optimal feeding on post-discharge weight gain, mortality, and time taken to regain birth weight. Moreover, the issue we examined has been largely neglected and has not undergone comprehensive study, lacking both sufficient scholarly focus, accurate investigation and has hitherto been insufficiently prioritized.

## Methods

### Study design

This was a single-center prospective observational study.

### Study site and setting

The study was conducted in the Special Care Unit (SCU) of Kawempe Hospital— a national referral hospital located in Kawempe Division, Kampala District, which lies at 00°21'43”N 32°33'41”E. It offers maternal and child health services, including obstetrics and gynecology, general pediatrics, laboratory services, pharmacy, and radiology services. It has a neonatology special care unit and also serves as a teaching hospital.

On average, the SCU admits 4,600 babies annually, of which more than 50% are very low birth weight infants. Both in-born and out-born babies are admitted. Despite having an official capacity of 60, with 13 baby cots, 50 incubators, 25 radiant warmers, and four beds, the SCU often accommodates over 120 babies at a single time.

The unit has separate preterm and term infant sections run by specialist Neonatologists, Pediatricians, and medical officers. Care within the SCU is all-encompassing, including providing warmth through incubators and radiant warmers, pulse-oximetry, phototherapy, bubble Continuous Positive Airway Pressure (CPAP), and enteral feeds. Very low birth weight infants are primarily nourished with Expressed Breast Milk (EBM), typically initiated on Day 3 of life at 1 ml (approximately 10 mls/kg/day). Parenteral nutrition is not available.

At Kawempe Hospital's Special Care Unit, enteral feeding for infants usually starts between the second and fourth day of life, after a small 1–2 ml trial feed is tolerated. Feeds are not based on weight and rarely begin on day one. The attending physicians determine and set the volume based on the infant's clinical status and tolerance, increasing it by 1 ml daily to the two-hourly feeds. Discharge typically occurs at 10 ml per feed, or sooner if condition allows. Despite this protocol, the feeding infants receive is considered sub-optimal.

They are discharged from the SCU when they are comfortably taking enteral feeds amounting to 10 mls every 2 h and are physiologically stable with normal axillary temperature, off oxygen, off intravenous fluids, apnea-free, and when the primary caretaker is comfortable taking care of the child at home. Attainment of complete feeds, weight, and gestational age are generally not fully considered when making the decision to discharge.

### Study duration

The study was conducted over a span of four months, commencing in October 2019 and concluding in January 2020. Each participant was monitored until the occurrence of one of the following: attainment of their seventh day of life, unfortunate demise, or the event of discharge from the hospital, with the earliest of these events marking the cessation of the follow-up period.

### Study population and characteristics of participants

The study focused on a specific cohort within the study population, which was constituted by neonates classified as VLBW, with individual weights falling below 1,500 g but not less than 1,000 g. These infants were admitted into the SCU of Kawempe Hospital within the study period. The VLBW infants served as the primary subjects of the study. At the same time, their mothers or designated caregivers were engaged as the respondents, offering valuable insights into these neonates' care and health outcomes during the study duration.

### Inclusion and exclusion criteria

VLBW infants whose mothers gave informed consent to participate were included in the study. Infants were excluded if they had significant medical or surgical contraindications to initiating or advancing enteral feeding during the study period; examples include suspected or confirmed necrotizing enterocolitis (NEC Stage ≥ II), existing intestinal obstruction, severe hemodynamic instability requiring high-dose vasoactive support, or prolonged ileus following recent major abdominal surgery. Furthermore, infants with major congenital anomalies known to significantly impact feeding ability or intrinsic growth potential were excluded, such as unrepaired major gastrointestinal anomalies (e.g., gastroschisis, omphalocele, esophageal atresia/tracheo-oesophageal fistula, cleft palate with inability to suck), severe hemodynamically significant congenital heart disease, aneuploidies/ major chromosomal syndromes associated with growth restriction (e.g., Trisomy 13, or 18), or severe craniofacial anomalies precluding enteral intake.

### Sample size estimation

The sample size for this study was estimated using the formula for proportions in a finite population (22).

$$n=\frac{\left[DEFF*Np\;(1-p)\right]}{\left[\frac{d^{2}}{Z_{1-\propto /2}^{2}}(N-1)+p\;(1-p)\right]}$$

In determining the sample size, several key variables were considered. Firstly, a design effect of 1.0 was applied, reflecting the randomized nature of the sample selection. The population size under consideration was 2,500, representing the annual admissions of very low birth weight neonates. The prevalence rate, estimated at 52.6%, was derived from a precedent study conducted at Kenyatta Hospital, which focused on the feeding practices among VLBW infants (23). Furthermore, statistical considerations included a 5% significance level and a power of 80%, ensuring robustness in the study's findings. Lastly, accounting for a projected non-response rate of 10%, the final calculated sample size was established at 370 participants.

### Study procedure and processes

The registry provided a record of all infants admitted to the SCU in the previous 24 h. Kawempe Hospital serves as a national referral center, admitting patients from diverse regions across Uganda. As a result, the sampling of VLBW infants inherently included a randomized component due to the hospital's wide catchment area. However, for practical reasons, participant enrollment was conducted using convenience sampling, where all eligible VLBW infants specifically those with a birth weight under 1,500 g but not less than 1,000 g, admitted to the SCU within the preceding 24 h were consecutively enrolled until the required sample size was attained. This methodological decision was deliberately chosen to balance pragmatic clinical research constraints with scientific rigor. This approach enabled us to efficiently collect a sample that, while not strictly random in the statistical sense, still provided a reasonably representative cross-section of the VLBW infant population in Uganda.

In the initial 24 h after birth, the gestational age of the infants was assessed using the Ballard score chart by the attending medical officer, and the findings were recorded in their files. Mothers whose infants aligned with the inclusion criteria were informed of the study's objectives and procedures by the research assistants to ensure informed consent.

Subsequently, on the second day of life, infants were officially enrolled in the study, assigned a unique study number, and a color-coded tag was affixed to their file for easy reference. The primary respondent—generally the infant's mother—was then engaged in a discussion using a semi-structured, interviewer-administered questionnaire. In the case of multiple births (twins or triplets), a fair ballot system was employed to select a single infant for the study to prevent undue burden on the mother. This random selection avoided intra-mother clustering, ensuring that each observation represented an independent mother–infant dyad. We recognize that this design prevents direct evaluation of multiple gestation as a risk factor and discuss this in the Limitations. We also note that while this approach ensured feasibility, it precluded analysis of twin/triplet-specific feeding dynamics. Future studies should prioritize enrolling all multiples to explore these effects.

Each infant involved in the study underwent an examination for congenital anomalies. Their hospital charts were also scrutinized using a checklist to evaluate infant-specific factors such as gestational age, birth weight, Apgar score, and the nature of birth (singleton or multiple). The study participants were closely monitored until the seventh day of life. Infants unable to reach this milestone due to unfortunate circumstances such as death or early discharge were noted, and the study concluded on their specific exit day.

The Multi-Dimensional Scale of Perceived Social Support (MSPSS), adapted from Zimet (24), was employed to comprehensively assess maternal psychosocial support networks. We used the MSPSS because mothers' ability to remain in the ward every two hours—a pre-requisite for mother-delivered feeds depends heavily on transport, childcare, and emotional support from family and friends. The MSPSS has demonstrated predictive validity for breastfeeding practices in hospital-based neonatal cohorts in LMIC settings (25, 26). This validated 12-item instrument evaluates perceived social support across three domains: family, friends, and significant others. Participants responded to statements using a 7-point Likert scale ranging from 1 (“very strongly disagree”) to 7 (“very strongly agree”). Responses were averaged to generate a final score, with interpretation categorized as follows: low support (1.0–2.9), moderate support (3.0–5.0), and high support (5.1–7.0). This framework enabled systematic evaluation of mothers' psychosocial assistance systems, ensuring alignment with evidence-based thresholds for social support categorization. The tool's integration of closed-ended quantitative scoring and domain-specific insights allowed for a nuanced analysis of how social support networks influenced infant feeding practices in the study population.

Each day, the weights of the enrolled infants were measured using a Seca 354 m digital baby weighing scale and documented in grams on the data capture sheet. Infants were weighed without any clothing or diapers to minimize measurement error. This measurement routine was consistently conducted each morning before administering the 8:00 a.m. feeds.

In the SCU, mothers receive detailed instructions on administering nasogastric tube (NGT) feeds to their infants and are guided on the daily quantity of feeds as a routine protocol. All enteral feeds (including NGT feeds) were given exclusively by the mother; nursing staff did not substitute feedings in the mother's absence. Consequently, any two-hour interval without a maternal visit was recorded as zero volume. Maintaining a non-interventionist approach, the research team did not influence the type or quantity of feeds, nor any other aspect of the infants' care. Research assistants observed and recorded the volume of enteral feeds provided through the NGT by mothers, executing this task every two hours, both day and night.

Per the SCU's standard procedures, mothers are expected to visit the unit every two hours to feed and monitor their infants. Instances where feeds were recorded as zero on the chart included occasions when mothers did not visit the SCU at the specified two-hour intervals; infants who were exclusively on intravenous solutions, such as 10% IV Dextrose and IV Ringer's lactate, instead of initiating enteral feeds; and the quantity of breast milk consumed directly from breastfeeding, based on the observation that the amount ingested by VLBW infants is minimal due to their underdeveloped suckling reflex (10).

The study utilized (1) to evaluate the adequacy of the daily feed amounts. An infant was considered to be receiving sub-optimal feeding if they failed to achieve the total feed volume of 150 mls/kg/day by the seventh day of life. For infants who did not survive until Day 7, the WHO's recommended optimal feed volumes for the corresponding day of life were calculated, for instance, 10–20 mls/kg on Day 1 and 30–40 mls/kg on Day 2, as shown in Table 1.

**Table 1:** Typical cumulative target volumes for rapid advancement of feeds per kg/day (WHO 2011 guideline, rapid schedule for VLBW infants).

Day of life	Recommended enteral feeds (mls/per kg/day)
Day one	10–20
Day two	30–40
Day three	50–60
Day four	70–90
Day five	100–120
Day six	120–140
Day seven	150–170

(Adapted from the WHO guidelines on optimal feeding of low-birth-weight infants in low-and- middle-income countries, 2011) The guideline also outlines a ’slow advancement’ schedule (10–20 ml kg^−^¹ day^−^¹). Kawempe SCU follows the rapid schedule; therefore, infants not achieving the cumulative volumes above were categorized as sub-optimally fed.

Infants receiving lesser quantities than the WHO recommendations for a specific day were categorized as having sub-optimal feeding.

### Data management and statistical analyses

Data were entered into data collection tools using confidential patient identifiers. Files were verified for completeness and stored securely. Epidata 3.1 was used for electronic database entry with quality checks. The data underwent double entry and validation by the Principal Investigator. The final dataset was backed up and exported to STATA version 14.1 for analysis, ensuring confidentiality throughout the process.

The research utilized univariate and bivariate analysis to characterize the study sample. Frequencies and proportions represented categorical variables, whereas continuous variables were depicted through medians and interquartile ranges. Chi-square and rank sum tests assessed the relationship between variables and sub-optimal feeding. Before multivariate analysis, individual logistic regressions evaluated independent variables' associations. Variables meeting a *p*-value threshold of <0.2, not inducing multicollinearity, and devoid of sparse data were included in multivariate analysis, further detailed subsequently. Screening results are presented in Table 6; variables with *p* < 0.20 and Variance Inflation Factor **(**VIF) < 4 were entered into the multivariable model.

Our study employed multivariable logistic regression to identify independent factors linked with sub-optimal feeding, significant at *p* < 0.05 with a 95% confidence interval. This prospective cohort study employed logistic regression for multivariate analysis, given the time-point outcome on Day 7.

This approach followed univariate analysis findings, where the prevalence of sub-optimal feeding notably exceeded that of optimal feeding, necessitating logistic regression for more profound insight.

The rate of early weight change (commonly termed growth velocity) was considered a secondary outcome. It is defined as weight gain in grams per kilogram per day and was computed by dividing the weight at the initial stage by the birth weight, then further divided by the days from birth to that initial stage. Recognizing that very low birth weight infants may lose up to 10% of their birth weight in their first week, this metric in the early postnatal period primarily reflects the success in mitigating physiological weight loss rather than net tissue accretion. Sub-optimal rate of weight change (sub-optimal growth velocity) was classified as weight gain of less than 15–20 g/kg/day or weight loss exceeding 7%–10% within the first seven days. The correlation between sub-optimal feeding and this early weight change trajectory was initially identified using the Odds Ratio. The impact of sub-optimal feeding on achieving a sub-optimal rate of weight change was subsequently established by comparing the proportion of infants with sub-optimal feeding who achieved sub-optimal growth velocity with those who received optimal feeds.

## Results

### Study profile and descriptive characteristics of study participants

A total of 370 VLBW infants admitted to the Special Care Unit (SCU) participated in the study. Out of these, 124 infants, representing 33.5% of the total number, unfortunately, passed away. Meanwhile, 88 (23.8%) were discharged before the seventh day. The remaining 158 infants, constituting 42.7% of the total, were closely monitored until the seventh day of life. After the seventh day, these infants continued their stay in the SCU for further observation and care.

As depicted in Table 2, of the 370 recruited VLBW infants, 187 (50.5%) were male, thereby establishing a male: female gender ratio of 1:1. The median assessed gestational age was observed to be 32 weeks, with an Interquartile Range (IQR) of 30–33 weeks. A significant proportion of the infants, 34.6%, had birth weights between 1.00 and <1.25 kg. Furthermore, comorbidities such as Respiratory Distress Syndrome (RDS), anemia, and sepsis were found in 201 (54.3%) infants.

**Table 2:** Descriptive characteristics of VLBW infants admitted to the SCU.

Descriptive characteristic	Median
Birth order; median (IQR)	2 (1–3)
Estimated Gestation Age; median (IQR), weeks	32 (30–33)
Apgar 5 min; median (IQR)	8 (6–9)
Gestational-age group (Ballard)	Frequency (*n* = 370)
<30 weeks	62 (16.8%)
30–31 weeks	89 (24.1%)
32–33 weeks	156 (42.1%)
≥34 weeks	63 (17.0%)
Birth weight (kilograms)*	Frequency (*n* = 370)
1.00 to <1.25	128 (34.6%)
1.25 to <1.50	231 (62.4%)
Sex	
Female	183 (49.5%)
Male	187 (50.5%)
Type of birth	
Singleton	258 (69.7%)
Multiple	111 (30.3%)
Congenital malformations (polydactyly, syndactyly, Trisomy 21)	10 (2.7%)
Comorbidities (RDS, sepsis, anemia)	201 (54.3%)

IQR, interquartile range.

* Missing data- 11.

### Feeding characteristics of the study participants

Among the enrolled infants, 167 (45%) began receiving enteral feeds within 48 h after birth. Meanwhile, 101 (27.3%) started enteral feeds after the first 48 h of life. Notably, 102 (27.7%) did not receive any enteral feeds during the study. These 102 infants were administered intravenous (IV) fluids, specifically Dextrose 10% and Ringer's Lactate, as an alternative to enteral feeding (Table 3). The primary reasons for delayed initiation (>48 h) were infant clinical instability (e.g., severe respiratory distress requiring CPAP or oxygen support), postpartum maternal complications (delayed lactogenesis or C-section recovery), and occasionally delayed nasogastric tube placement. Infants who received no enteral feeds were those with medical contraindications to gut feeding (profound sepsis or hemodynamic instability) or whose mothers were unable to provide breastmilk throughout their stay and were therefore managed exclusively on IV fluids.

**Table 3:** Feeding characteristics of VLBW infants admitted to the SCU.

Feeding characteristic	Frequency (*n* = 370)
Initiation of enteral feeds	
≤48 h	167 (45.0%)
>48 h	101 (27.3%)
Not initiated	102 (27.7%)
Type of feeds received	
Breast milk	255 (68.9%)
Formula milk	4 (1.1%)
Both formula and breast milk	9 (2.3%)
No enteral feeds*	102 (27.7%)
Method of feeding**	
Breastfeeding	81 (21.9%)
Through the NGT (Gavage feeding)	101 (27.3%)
Both Through the NGT and breastfeeding	73 (19.7%)
Others***	4 (1.1%)

* Infants who did not receive enteral feeds received IV fluids, e.g., IV Dextrose 10% and IV Ringer's Lactate.

** *N* = 268 (only among those who received enteral feeds).

*** Other modes of feeding included using a syringe and using a spoon.

### Descriptive characteristics of the mothers of the VLBW infants

The infants' biological mothers were the primary respondents, 356 (96.2%) in the study (Table 4). The median age amongst this group was 26 years, with the interquartile range between 22 and 29 years. Interestingly, 42 (11.6%) were teenage mothers. A significant portion, 206 (60%) of the mothers had attended Antenatal care (ANC) fewer than three times.

**Table 4:** Characteristics of mothers to VLBW infants in the SCU.

Characteristic	Frequency (*n* = 370)
Age	
Teenage mother	42 (11.6%)
Non-teenage mother	319 (88.4%)
*Religion	
Christian	288 (77.8%)
Moslem	82 (22.2%)
*Occupation	
Employed	220 (60.4%)
Unemployed	144 (39.6%)
*Marital status	
Living with partner	300 (81.1%)
Single	70 (18.92%)
Education level	
Primary	121 (33.24%)
Secondary	191 (51.62%)
Tertiary	56 (15.14%)
Average monthly income (USD)	
<14	74 (20.6%)
14–55	107 (29.8%)
>55–138	151 (42.1%)
>138	27 (7.5%)
Parity	
Median (IQR)	2 (1–3)
Frequency of ANC attendance	
<3	206 (60.0%)
≥3	140 (40.0%)
*Illnesses during pregnancy, e.g., malaria, UTI*	
Yes	107 (28.9)
Chronic illnesses in the mother, e.g., HIV, SCA*	
Yes	61 (16.5%)
Mode of delivery	
Caesarian Section	71 (19.2%)
SVD	299 (80.8%)
Ever heard about VLBW infants	
Yes	302 (81.6%)
Attitude towards their VLBW infant	
I feel good/happy/excited	147 (39.7%)
I feel overwhelmed/scared	176 (47.5%)
Not sure about how I feel	47 (4.6%)
*Confidence in feeding VLBW infant	
Yes	232 (62.7%)
*Satisfaction with instructions on feeding	
Satisfied*	231 (62.4%)
Not satisfied	139 (37.5%)

* Satisfied = “somewhat satisfied”, “satisfied”, and “very satisfied”.

Missing; Age-9, Occupation-6, Education level-11, No. of ANC visits-24.

ANC, antenatal care; UTI, urinary tract infection; SCA, sickle cell anemia; IQR, inter-quartile range; SVD, spontaneous vertex delivery.

### Proportion of sub-optimal feeding among VLBW infants followed up in the SCU

**Figure 1:**
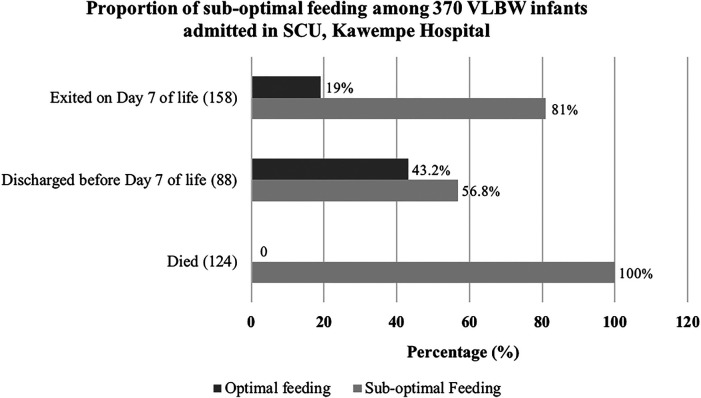
Proportion of sub-optimal feeding among VLBW infants admitted to the SCU.

The study revealed that 333 (90%) VLBW infants had sub-optimal feeding. Within this group, 102 infants (30.6%) received no enteral feeds, relying solely on intravenous solutions like 10% Dextrose and Ringers' Lactate. Of the 158 infants who left the study on the seventh day, 128 (81%) had sub-optimal feeding. Among the 88 infants discharged before the seventh day, 50 (56.8%) faced the same issue. Notably, all 124 infants who passed away during the study had sub-optimal feeding. Figure 1 below further elucidates the results.

### Trends in sub-optimal feeding among VLBW infants followed up in the SCU over seven days

**Figure 2:**
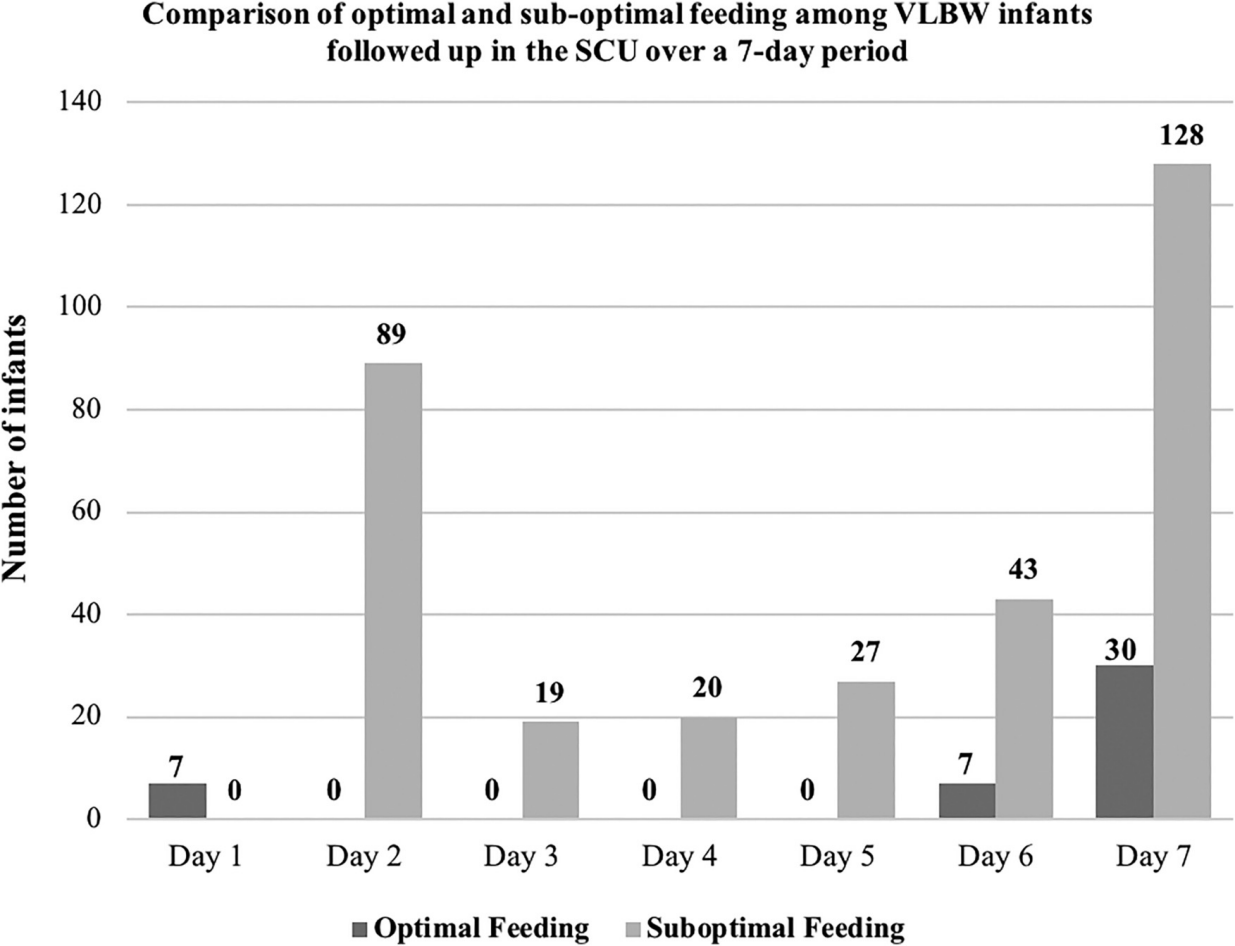
Comparison of optimal and sub-optimal feeding among VLBW infants followed up in the SCU over seven days.

Of the 333, the number of infants who received sub-optimal feeding exponentially rose from day 1, peaking on day 2 of life. This declined between the third and sixth day before seeing another surge on the seventh day of life. Conversely, the number of infants fed optimally began to climb on the sixth day, reaching its peak on the seventh day. Figure 2 below summarizes the results.

### Bivariate analysis of the association between maternal and infant factors and sub-optimal feeding among very low birth weight infants in the SCU

During the bivariate analysis, with a confidence level of 95%, several independent variables were identified to have a significant correlation with sub-optimal feeding. These variables included birthweight (*p*-value 0.007), initiation of feeds (*p*-value 0.002), other comorbidities (*p*-value 0.01), average monthly income (*p*-value 0.039), and the mothers' final social support score (*p*-value 0.002). The lack of significance in the 1.0–1.25 kg cohort may reflect stricter adherence to feeding protocols for critically vulnerable infants, whereas the 1.25–1.5 kg group's heterogeneity in clinical stability likely contributed to observable associations with sub-optimal feeding.

Infants not initiated on enteral feeds were 10.07 times more likely to experience sub-optimal feeding than those who started feeding within the first 48 h of life. Moreover, infants whose mothers received moderate social support were 3.14 times more prone to sub-optimal feeding than those with mothers who had high social support. Details are depicted in Table 5.

**Table 5:** Bivariate analysis showing the association between independent variables and sub-optimal feeding among VLBW infants in the SCU.

Characteristics	Sub-optimal feeding *n* (%)	OR (95% CI)	*p* value
Birth weight			
1 to <1.25 kg	118 (35.98)	1.00	—
1.25 to 1.50 kg	199 (60.67)	0.26 (0.10–0.69)	**0**.**007**
Initiation of feeds			
≤48 h	139 (53.67)	1.00	—
>48 h	94 (36.29)	2.71 (1.13–6.45)	**0**.**025**
Not initiated	26 (10.04)	10.07 (2.35–43.26)	**0**.**002**
Other comorbidities (RDS, Sepsis, Anemia)			
Yes (reference)	189 (57.62)	1.00	—
No	139 (42.38)	0.38 (0.18–0.80)	**0**.**010**
Religion			
Christian	252 (78.75)	1.00	—
Moslem	68 (21.25)	0.44 (0.21–0.91)	**0**.**026**
Average monthly income (USD)			
<14	70 (21.67)	1.00	—
14–55	90 (27.86)	0.30 (0.10–0.94)	**0**.**039**
>55–138	139 (43.03)	0.66 (0.21–2.13)	0.488
>138	24 (7.43)	0.46 (0.10–2.19)	0.328
Attitude towards VLBW infant			
I feel good/happy	128 (39.38)	1.00	—
I do not feel good/happy	197 (60.62)	1.62 (0.82–3.21)	0.163
No. of times mother attended ANC.			
<3	189 (61.17)	1.00	
≥3	120 (38.83)	0.54 (0.27–1.07)	0.078
Final social support score			
Low	19 (5.76)	2.24 (0.48–10.57)	0.307
Moderate	239 (72.42)	3.14 (1.54–6.40)	**0**.**002**
High	72 (21.82)	1.00	

Reference category is 1.0 ≤ 1.25 kg; ORs refer to comparisons against that group. ORs <1 indicate that absence of comorbidities is protective, implying that presence of comorbidities increases odds of sub-optimal feeding.

The bolded *p*-values indicate independent variables identified to have a significant correlation with sub-optimal feeding.

Table 6 summarizes univariate screening results for all candidate predictors. Initiation of feeds, birth-weight group, gestational age group (32–33 weeks and ≥34 weeks vs. <30 weeks), other comorbidities, ANC visits, household income (14–55 USD vs. <14 USD), and maternal social support (moderate vs. high) met the inclusion criteria (*p* < 0.20) and were taken forward for multivariate analysis. Feed type, maternal age, sex, multiple birth, maternal education, and low social support were excluded because *p* > 0.20. Birth-weight group was subsequently dropped from the multivariate model due to high collinearity with gestational age group (Variance Inflation Factor > 4).

**Table 6:** Univariate logistic regression screening of candidate predictors for sub-optimal feeding.

Variable (category)	Reference category	Unadjusted OR (95% CI)	*p*-value	Entered MV model
Gestational-age group				
30–31 w vs. <30 w	<30 w	0.65 (0.30–1.41)	0.270	No
32–33 w vs. <30 w	<30 w	0.52 (0.23–1.14)	0.100	Yes
≥34 w vs. <30 w	<30 w	0.40 (0.15–1.08)	0.070	Yes
Birth-weight group				
1.25–1.50 kg vs. 1.00–1.25 kg	1.00–1.25 kg	0.26 (0.10–0.69)	0.007	No*
Sex (male vs. female)	Female	1.12 (0.71–1.77)	0.630	No
Multiple birth (yes vs. singleton)	Singleton	1.33 (0.79–2.25)	0.28	No
Other comorbidities (no vs. yes)	Yes	0.38 (0.18–0.80)	0.010	Yes
Initiation of feeds				
>48 h vs. ≤48 h	≤48 h	2.71 (1.13–6.45)	0.025	Yes
Not initiated vs. ≤48 h	≤48 h	10.07 (2.35–43.26)	0.002	Yes
Feed type				
Formula/mixed vs. breast milk	Breast milk	1.12 (0.48–2.64)	0.790	No
Maternal age ≥20 yrs vs. <20 yrs	<20 y	0.91 (0.44–1.86)	0.790	No
ANC visits ≥3 vs. <3	<3	0.54 (0.27–1.07)	0.080	Yes
Household income				
14–55 USD vs. <14 USD	<14 USD	0.30 (0.10–0.94)	0.039	Yes
>55–138 USD vs. <14 USD	<14 USD	0.66 (0.21–2.13)	0.488	No
>138 USD vs. <14 USD	<14 USD	0.46 (0.10–2.19)	0.328	No
Maternal education				
Secondary vs. primary	Primary	0.93 (0.58–1.50)	0.770	No
Tertiary vs. primary	Primary	0.88 (0.41–1.89)	0.730	No
Final social support score				
Low vs. high	High	2.24 (0.48–10.57)	0.307	No
Moderate vs. high	High	3.14 (1.54–6.40)	0.002	Yes

* Birth-weight group is excluded from multivariable model because of high collinearity with gestational-age group (VIF > 4). Variables meeting the screening rule (*p* < 0.20 and variance-inflation factor < 4) are flagged “Yes” in the final column and were carried forward to the multivariable model (Table 7).

Gestational-age group did not show a statistically significant association with sub-optimal feeding for the 30–31 week category compared to <30 weeks (OR 0.65, 95% CI 0.30–1.41, *p* = 0.27), while the 32–33 week (OR: 0.52, 95% CI: 0.23–1.14, *p* = 0.10) and ≥34 week categories (OR: 0.40, 95% CI: 0.15–1.08, *p* = 0.07) showed trends toward significance and were included in the multivariate model (Table 6).

Feed type (exclusive breast milk vs. formula/mixed feeding) was not associated with sub-optimal feeding (OR: 1.12, 95% CI: 0.48–2.64, *p* = 0.79) and therefore was not retained for multivariate analysis.

Maternal education level was not associated with sub-optimal feeding (secondary vs. primary: OR: 0.93, 95% CI: 0.58–1.50, *p* = 0.77; tertiary vs. primary: OR: 0.88, 95% CI: 0.41–1.89, *p* = 0.73) and therefore was excluded from the multivariate model.

### Multivariate analysis of factors associated with sub-optimal feeding among VLBW infants followed up in the SCU

In multivariable logistic regression analysis, lack of initiation of enteral feeds and moderate maternal social support remained independently associated with sub-optimal feeding (Table 7).

**Table 7:** Multivariate logistic regression showing factors independently associated with sub-optimal feeding among VLBW infants in the SCU.

Characteristics	Unadjusted OR (95% CI)	*p* value	Adjusted OR (95% CI)	*p* value
Birth weight				
1 to <1.25 kg	1.00	—	1.00	—
1.25 to 1.50 kg	0.26 (0.10–0.69)	0.007	0.51 (0.17–1.54)	0.230
Initiation of feeds				
≤48 h	1.00	—	1.00	—
>48 h	2.71 (1.13–6.45)	0.025	2.18 (0.79–6.04)	0.132
Not initiated	10.07 (2.35–43.26)	0.002	11.03 (1.34–90.77)	**0**.**026**
Other comorbidities (RDS, Sepsis, Anemia)				
Yes	1.00	—	1.00	—
No	0.38 (0.18–0.80)	0.010	1.13 (0.49–2.57)	0.223
Religion				
Christian	1.00	—	1.00	—
Moslem	0.44 (0.21–0.91)	0.026	0.45 (0.19–1.11)	0.083
Average monthly income (USD)				
<14	1.00	—	1.00	—
14–55	0.30 (0.10–0.94)	0.039	0.48 (0.13–1.68)	0.248
>55–138	0.66 (0.21–2.13)	0.488	1.24 (0.33–4.66)	0.747
>138	0.46 (0.10–2.19)	0.328	1.11 (0.19–6.40)	0.906
Attitude towards VLBW infant				
I feel good/happy	1.00	—	1.00	—
I don’t feel good/happy	1.62 (0.82–3.21)	0.163	1.13 (0.49–2.57)	0.779
No. of times mother attended ANC.				
<3	1.00	—	1.00	—
≥3	0.54 (0.27–1.07)	0.078	0.71 (0.31–1.61)	0.412
Final social score				
Low	2.24 (0.48–10.57)	0.307	1.40 (0.22–8.78)	0.717
Moderate	3.14 (1.54–6.40)	0.002	2.78 (1.14–6.82)	**0**.**025**
High	1.00	—	1.00	—

Only variables meeting screening criteria (*p* < 0.20) from Table 6 were included in the multivariate model.

Bolded *p*-values indicate statistical significance (*p* < 0.05).

An infant who did not start on enteral feeds was 11.03 times [AOR = 11.03, 95%, CI (1.34 to 90.77)] more prone to have sub-optimal feeding than one who started within the first 48 h of life. An infant with a mother with moderate social support was 2.78 times [AOR = 2.78, 95%, CI (1.14 to 6.82)] more likely to have sub-optimal feeding than one with a mother with high social support. Although the point estimate for low maternal support also suggested increased odds (AOR 1.40; 95% CI: 0.22–8.78), this did not reach statistical significance, likely reflecting the small number of mothers in this category and resultant imprecision. Factors significantly associated with sub-optimal feeding at the bivariate analysis level but not at the multivariate level included birth weight, other comorbidities, religion, and the average household monthly income. The results are detailed in Table 7.

### Effect of sub-optimal feeding on the early rate of weight change (growth velocity) of VLBW infants followed up in the SCU

**Figure 3:**
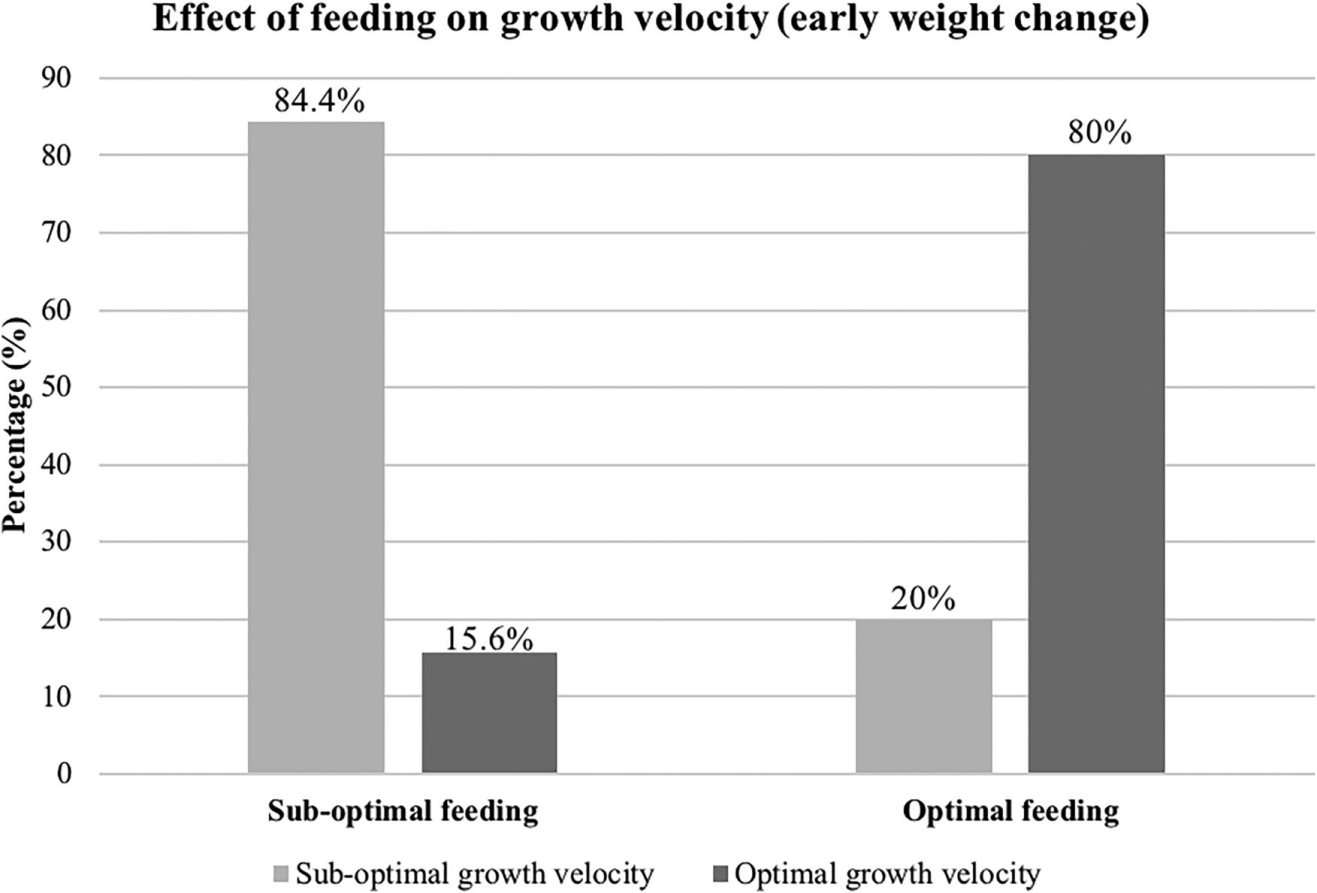
Effect of sub-optimal feeding on early weight-trajectory.

The study revealed that sub-optimal feeding was significantly associated with a sub-optimal early rate of weight change (growth velocity), primarily reflecting greater initial weight loss or failure to stabilize weight adequately. The odds of an infant with sub-optimal feeding having a sub-optimal rate of early weight change (meeting the criteria defined above) was 6.81 times [OR = 6.81, 95%, CI (2.74 to 16.97)] that of an infant with optimal feeding having sub-optimal growth velocity. Of the 158 infants who exited the study on Day 7 of life, 128 (81%) had sub-optimal feeding. Among these, 108 (84.4%) achieved sub-optimal growth velocity. Conversely, among the 30 infants who received optimal feeding, 6 (20%) achieved sub-optimal growth velocity, as depicted in Figure 3.

## Discussion

Our study revealed an alarming 90% rate of sub-optimal feeding among Very Low Birth Weight infants in Kawempe Hospital's Special Care Unit. This high rate can be attributed to a variety of factors.

A key finding of this study is the delayed initiation of enteral feeds, a crucial factor in the early nutrition of VLBW infants. Less than half (45%) of the infants in this setting were started on enteral feeds within the critical first 48 h, a stark contrast to optimal practices.

This delay was often due to unawareness and post-partum recovery complications resulting in insufficient breast milk production by the mother. Additionally, the underdeveloped suckling reflex of VLBW infants resulted in negligible breastmilk intake, a factor that was overlooked and not recorded (9).

A previous prospective study by Were et al. demonstrated a significantly lower rate (52.6%) of sub-optimal feeding among VLBW infants in Kenyatta Hospital (23). The discrepancy can be attributed to different methods of feed recording and a more extended follow-up period for infants in the Kenyatta Hospital study, allowing mothers more time to stabilize and produce adequate breastmilk. This disparity underscores the need for consistent and accurate tracking of neonatal feeding practices.

In our study, the key contributors to sub-optimal feeding were the delay in initiating enteral feeds and the lack of substantial social support for mothers. Comorbidities in infants, lack of NGT *in situ*, poor breastmilk flow due to maternal ill health, stress, recovery, or lack of information on feed initiation could account for the lack of feed initiation in almost a quarter of the infants. In addition, because breastmilk production physiologically peaks on day 9 (9, 27), the mothers could have produced optimal amounts of breastmilk after their infants had exited the study. Furthermore, our study revealed that 45% of infants were initiated on enteral feeds within the first 48 h, a figure significantly lower than the 80.1% observed in a study conducted at Kiwoko Hospital (16). This discrepancy can be attributed to the inclusion of preterm infants with a birth weight greater than 1,500 g in the Kiwoko study, who are more likely to be promptly initiated on enteral feeds, unlike our study, which focused on infants with birth weight less than 1,500 g. In this study, as in many others, the timing of starting enteral feeds was a crucial factor. Infants who did not begin feeding immediately were 11.03 times more likely to experience sub-optimal feeding than those who started feeding within the first 48 h of life. These findings concur with the WHO recommendation that starting enteral feeds within the first 48 h of life enables VLBW infants to achieve optimal feeding (1). The Kiwoko Hospital study by Nakubulwa et al. revealed that infants started on enteral feeds after 48 h were 3.7 times more likely to have post-natal growth failure than those started within the first 48 h (16). A related study conducted by Namiiro in the same environment found a significant link between delayed breast milk initiation (beyond 48 h) and the inability of infants to regain their birth weight within 21 days of age (28).

The study utilized the multi-dimensional social support score developed by (24). Our analysis revealed that mothers reporting moderate social support were 2.78 times more likely to receive sub-optimal feeds (AOR = 2.78) compared to those with high social support. This finding emphasizes the importance of a strong support system, potentially by reducing maternal stress and facilitating better care practices, aligning with studies showing that perceived social support's impact is non-linear and context-dependent (25, 26). Interestingly, while mothers with low social support also showed a trend towards increased risk (AOR = 1.40) compared to the high support group, this association did not reach statistical significance. This is likely attributable to the small size of the “Low Support” subgroup (*n* = 19, 5.8% of participants) in our study, which limited the statistical power to detect a significant effect and resulted in a wide confidence interval (95% CI 0.22–8.78). The lack of association with low social support may also suggest that mothers with very low support accessed alternative resources (e.g., hospital staff, community programs) to compensate. The larger size of the “Moderate Support” group (*n* = 239, 72.4%) provided sufficient power to identify its significant association with sub-optimal feeding. This highlights that lacking high levels of support, even if support is perceived as “moderate”, remains a critical risk factor in this context.

In our study, less than a quarter (25%) of the infants receiving sub-optimal feeding showed an optimal rate of early weight change (i.e., meeting the defined criteria for acceptable weight loss/gain) by day 7. In contrast, nearly all infants with optimal feeding achieved this growth metric. Furthermore, the chances of a sub-optimally fed infant experiencing a sub-optimal rate of early weight change are 6.81 times higher than an optimally fed infant (9, 11). This statistic reinforces the critical importance of optimal feeding in mitigating early catabolism and establishing a favorable weight trajectory achieving healthy growth rates among VLBW infants by day 7. Infants require the right balance of fats, proteins, and carbohydrates to sustain basal metabolic needs and grow (9, 11). Failure to provide these have detrimental effects on growth. VLBW infants are delivered in a nutritionally disadvantaged state, and failure to meet their nutritional needs predisposes them to a poorer early weight trajectory. To affirm this, studies by Nakubulwa et al., and Namiiro et al. demonstrated that sub-optimally fed infants are more likely to have growth restriction in the post-natal period, take longer to regain their birth weight and have higher chances of post-natal mortality (16, 28). Sub-optimal feeding also influenced mortality rates, with a third (33.5%) of the infants in our study not surviving.

In our study, some factors surprisingly did not contribute significantly to sub-optimal feeding amongst VLBW infants. These include infant comorbidities, gestational age, type of feeds, and the mother's education level. Despite other studies highlighting the role of infant comorbidities in feeding challenges, this was not the case in our analysis, suggesting these might have acted as confounders in multivariate analysis.

In agreement with Nakubulwa et al. (16), in our study, gestational age was not significantly associated with post-natal growth struggles (Table 6), although other studies have reported an effect on neonatal outcomes (29, 30). However, it is worth noting that smaller preterm infants have been shown to lose more weight and require a longer period to regain it (9).

Interestingly, the type of enteral feed provided (e.g., exclusive breast milk, mixed feeding, formula) did not emerge as an independent predictor significantly associated with our primary outcome of sub-optimal feeding, which was defined based on achieving target daily volumes. This finding in our study appears somewhat counterintuitive, given that established neonatal nutritional guidelines emphasize the importance of feed composition for adequate growth velocity in VLBW infants. Specifically, recommendations strongly support the use of multi-component human milk fortifiers or nutrient-dense preterm formulas compared to unfortified human milk alone to meet the high metabolic demands and promote optimal growth (23, 31). It is worth noting that robust clinical trial evidence for fortification's impact on long-term outcomes remains somewhat limited, with recommendations often based on nutrient accretion data (32). The lack of a significant association between feed type and volume-based feeding success in our specific study context could potentially be due to several factors, including: our primary outcome definition not directly assessing growth velocity or nutrient intake adequacy, the relatively short 7-day follow-up period, insufficient statistical power to detect differences related to feed type for this specific outcome, or inconsistent application or availability of fortification within the unit during the study period.

In our cohort, maternal education level was not significantly associated with sub-optimal feeding (Table 6), although other studies have reported it as a significant determinant of neonatal outcomes. This finding contradicts a study in Italy, where lower educational levels were linked to poorer outcomes for VLBW infants (24). A meta-analysis by (29) also reported it as a significant determinant.

Our study challenges some commonly held beliefs and opens avenues for more research to holistically understand the peculiarities of feeding VLBW infants in our setting.

### Limitations of the study

This study, conducted to evaluate the prevalence, contributing factors, and impact of sub-optimal feeding on the growth trajectories of VLBW infants in Kawempe Hospital's Special Care Unit, is not exempt from certain limitations and constraints. Acknowledging these limitations is crucial for an impartial evaluation of the research outcomes and provides valuable insights for future scholars to enhance their methodological approaches in this field.

Weight measurements could have been erroneously taken, leading to misclassification. To mitigate potential errors, the study employed a calibrated weighing scale and utilized research assistants skilled in infant weight measurement, coupled with training on standardized procedures to ensure accuracy. Direct observation of the mothers could have introduced social desirability bias, resulting in mothers altering their behavior, knowing they are observed. This was minimized through a discreet observational approach and monitoring by the research assistants. The study did not examine or explore health system factors due to resource constraints. Another critical limitation of this study was the brief seven-day follow-up period, which restricted the ability to study the full effect of sub-optimal feeding on growth velocity.

The small sample size of mothers with low social support (*n* = 19) limited our ability to detect significant associations in this subgroup.

Perceived social support was measured once during hospitalization; the score could change after discharge, which may introduce misclassification bias.

Additionally, the study's protocol of selecting only one infant from multiple births (via fair ballot, to reduce maternal burden) limits the ability to analyze the specific impact of multiple gestation on feeding outcomes within this dataset. By enrolling only one infant per multiple-birth set we were unable to study the specific impact of multiple gestation on feeding outcomes.

Furthermore, while the study identified significant delays in feeding initiation (>48 h or not initiated at all) as a key issue, its scope did not include the systematic collection of the specific clinical reasons documented for each individual infant's feeding plan, precluding a detailed analysis of the proximate causes for these specific feeding patterns within this dataset.

Finally, the study focused solely on weight-based growth velocity and did not capture other anthropometric measures such as length, head circumference, or skinfold thickness which limited our capacity to evaluate overall growth patterns and body composition. These parameters are critical for comprehensively assessing growth and neurodevelopmental outcomes in VLBW infants (26, 27). Their exclusion limits our understanding of the multidimensional impact of sub-optimal feeding on physical and neurological development.

## Conclusions

This cohort study documents an alarmingly high prevalence (90%) of sub-optimal enteral feeding among very-low-birth-weight infants admitted to the Kawempe Hospital Special Care Unit. Failure to initiate feeds within the first 48 h (or not at all) was the strongest independent predictor of sub-optimal intake (AOR ≈ 11.03), and infants whose mothers reported only moderate social support were also at higher risk (AOR ≈ 2.78). Sub-optimal feeding was associated with greater early post-natal weight loss by Day 7 and increased mortality, underscoring the clinical impact of inadequate nutrient delivery during the initial week of life. These findings highlight two actionable determinants—timely feed initiation and maternal support that warrant close attention to improve overall neonatal outcomes in similar low-resource neonatal settings. Improving these practices is critical for enhancing survival and neonatal outcomes in this vulnerable population.

### Implications and recommendations

The 90% prevalence of sub-optimal enteral feeding among VLBW infants at Kawempe SCU signals a systemic gap that extends beyond this single facility. This gap has severe consequences, as demonstrated by the strong association between sub-optimal feeding and poor early weight outcomes (OR = 6.81) and the overall high mortality observed in the cohort. Three inter-locking domains merit immediate attention.

1. **Clinical protocols**: Rigidly initiating enteral feeds within the first 48 h and documenting cumulative volumes against WHO rapid advancement targets should become a non-negotiable standard of care. This requires overcoming common barriers including infant instability, maternal postpartum complications, delayed lactogenesis, and procedural difficulties. Implementing a bedside “feed monitor chart” that flags missed two-hour feeds in real time will allow nurses to prompt maternal visits or, when clinically indicated, administer expressed breastmilk themselves. Standardized protocols for initiating minimal enteral nutrition even in less stable infants should be considered where appropriate. Early breast milk fortification or pre-term formula should be considered for infants who cannot meet daily volume goals by day three.
2. **Maternal support systems**: Moderate perceived social support—affecting nearly three quarters of mothers—doubled the odds of sub-optimal feeding. Hospitals in similar low-resource settings should pilot low-cost interventions such as on-site lactation lounges, transport stipends, and peer counsellor programs run by mothers of previously admitted pre-term infants. Embedding a one-item social support screen into admission paperwork would allow staff to identify high-risk mothers on day one and connect them to targeted assistance.

#### Health system and research priorities

At policy level, national neonatal guidelines should incorporate explicit feed initiation timelines and mandate routine social support assessment. District health offices can use our prevalence estimate (90%) and effect size for delayed feeds (AOR 11.0) as benchmarks for quality improvement indicators. Future multi-centre studies should (i) enroll complete sets of multiple births, (ii) capture head circumference and length to evaluate global growth, (iii) conduct root cause analyses to systematically identify specific reasons for delayed feed initiation, and (iv) test bundled interventions combining rapid feed initiation with structured maternal support packages on longer-term neurodevelopment outcomes.

## Data Availability

The raw data supporting the conclusions of this article will be made available by the authors, without undue reservation.
